# Image Stitching Based on Nonrigid Warping for Urban Scene

**DOI:** 10.3390/s20247050

**Published:** 2020-12-09

**Authors:** Lixia Deng, Xiuxiao Yuan, Cailong Deng, Jun Chen, Yang Cai

**Affiliations:** School of Remote Sensing and Information Engineering, Wuhan University, Wuhan 430079, China; denglixia@whu.edu.cn (L.D.); dcl@whu.edu.cn (C.D.); jchen_cug@whu.edu.cn (J.C.); Y.Tsai@whu.edu.cn (Y.C.)

**Keywords:** image alignment, image stitching, nonrigid warping, parallax-tolerant, urban scene

## Abstract

Image stitching based on a global alignment model is widely used in computer vision. However, the resulting stitched image may look blurry or ghosted due to parallax. To solve this problem, we propose a parallax-tolerant image stitching method based on nonrigid warping in this paper. Given a group of putative feature correspondences between overlapping images, we first use a semiparametric function fitting, which introduces a motion coherence constraint to remove outliers. Then, the input images are warped according to a nonrigid warp model based on Gaussian radial basis functions. The nonrigid warping is a kind of elastic deformation that is flexible and smooth enough to eliminate moderate parallax errors. This leads to high-precision alignment in the overlapped region. For the nonoverlapping region, we use a rigid similarity model to reduce distortion. Through effective transition, the nonrigid warping of the overlapped region and the rigid warping of the nonoverlapping region can be used jointly. Our method can obtain more accurate local alignment while maintaining the overall shape of the image. Experimental results on several challenging data sets for urban scene show that the proposed approach is better than state-of-the-art approaches in both qualitative and quantitative indicators.

## 1. Introduction

Urban scene images acquired by optical sensors have a wide range of applications in urban informatization, such as environmental monitoring, road planning, street-view map production, and 3D urban reconstruction [1,2,3,4]. Due to the limitations of the camera’s viewing angle and shooting distance, the area covered by a single image is small. Therefore, it is necessary to use image stitching technology to expand the coverage of the image and obtain more information from the target area.

Image stitching is a process of merging a group of images into a larger image with a wider field-of-view of the scene. It can usually be solved by aligning images based on their common features. The sparsely scattered, distinctive, and well-localized key points provided by the sparse feature matchers have been widely used in image correlation. Although the sparse feature matchers lack the corresponding density provided by the dense matching method [5], it can provide the advantages of wide baseline, fast speed, and unlimited data types [6]. Most of the image alignment algorithms aim to find a two-dimensional global warp model between two overlapped images, such as similarity, affine, and homography. A fine global warp minimizes the total registration error instead of exactly aligning all the features; therefore, it is robust but not sufficient to adapt to all scenes. In computer vision, homography model is widely used to describe the projection relationship within an image pair. However, it only works when the scene is planar or the camera undergoes pure rotation [7]. When the parallax is too large to be ignored, unexpected “ghosts” will appear in the stitched images aligned by a single global warp model. For street view images in urban scenes that contain rich objects and complex depth changes, it is necessary to consider image stitching methods that can handle parallax.

In order to improve alignment accuracy in the presence of parallax, scholars have conducted extensive work in the field of computer vision. In summary, the existing methods are based on the following three ideas. One of the ideas is to search for seam lines to bypass the misalignment in the overlapped region [8,9,10,11]. Seam-based methods usually have a high computational cost and are more suitable for the situation in which there are obvious foreground objects and background in the images. Another idea is to adopt multiple transformation models [12,13,14]. The third idea is to use surface fitting to deal with the parallax on the two-dimensional image [15,16,17]. Actually, it is believed for the last two ideas that different regions of the image should utilize different warp models because, in the image obtained by pinhole imaging, objects closer to the shooting center will have greater parallax. Therefore, they all tried to find an image alignment model that changes with space.

In addition, we noticed that the quality of feature matching directly affects the stitching quality. Most image alignment approaches employed the Random Sample Consensus (RANSAC) algorithm [18] to remove outliers of the matched features, and a global transformation usually serves as the minimal solver of RANSAC. There are contradictions between global RANSAC and spatially varying alignment: (1) If the threshold is too small, inliers might be rejected because they do not conform to the global transformation used by the RANSAC method, which is not conducive to local alignment; (2) If the threshold is too large, outliers might be preserved and lead to a poor stitching result. Good feature correspondences can refine the alignment, and a good alignment can verify the existing correspondences. Therefore, we need a more flexible feature match refinement method which can preserve spatially varying projection of the feature points.

In this paper, we propose a parallax-tolerant image stitching method that follows the idea of spatially varying alignment. Our goal is to find a good feature correspondence and, at the same time, determine a fine warp model to reduce registration errors. First, we establish a new feature mapping relationship base on semiparametric fitting. The semiparametric function includes a smoothness constraint based on the Motion Coherence Theory (MCT) [19], which provides greater flexibility for finding good feature maps through rough feature correspondence. Features that do not conform to the fitting functions are regarded as outliers. This idea is inspired by Lin et al. [20,21]; they used a complex smooth function to fit the feature correspondence and deal with the piecewise noises that RANSAC cannot handle. We design a smoothing function that is more suitable for two-dimensional plane warping and stitching. The advantage is that the alignment model can be directly derived from the feature correspondence. Then, we obtain a nonrigid warping based on the Gaussian radial basis function (GRBF) to eliminate misalignments in the overlapped region. Compared with TPS, GRBF is more suitable for local deformation [22]. Homography is a warping from one two-dimensional plane to another two-dimensional plane, while the nonrigid warp is more like performing a three-dimensional surface fitting first and then projecting onto a two-dimensional plane. Therefore, we can eliminate parallax errors which may be left by a single homography warping. Finally, we gradually change the nonrigid warp to the global homography warp to reduce unnecessary distortion in the nonoverlapping region. Meanwhile, a grid-based interpolation calculation method is used to improve efficiency. Experiments on several challenging image sets prove that our method can effectively reduce the projection errors, and can be well combined with other global methods.

## 2. Related Works

### 2.1. Feature Matching

In feature-based image stitching, feature matching is an important foundation. Lowe and David [23] proposed a sparse feature descriptor known as scale-invariant feature transform (SIFT), which is invariant to image translation, rotation, and scale. It is also robust to addition of noise, affine distortion, and changes in illumination [24]. Numerous studies have shown that the SIFT is the most widely used feature descriptor in image stitching and the performance has been demonstrated [25]. After preliminary matching based on feature descriptors, RANSAC was usually used to eliminate outliers based on the geometric relationship between images.

Among the researches we have investigated, Zhang et al. [26] introduced an outlier rejection method based on local homography to remove incorrect feature matchings; this method can only be used under the framework of the APAP method and is not universal. Guo et al. [27] assumed that the scene contains two planes. First, they found matches on one of the planes using the RANSAC method; then, they found matches on the other plane from the remaining points at the adjacent frame. Similar to DHW, this method is only suitable for specific scenes. Chen et al. introduced a nonrigid matching algorithm based on vector field consensus (VFC) [28] to the mosaic system for generating accurate feature matching [29]. In most methods other than global warping, the model fitted by RANSAC and the model used for alignment are relatively independent. Therefore, the retained feature points cannot help to optimize the stitching field.

### 2.2. Parallax-Tolerant Image Stitching

Gao et al. [8] estimated multiple warps from multiple sets of features, then used the quality of the seam line to evaluate the alignment performance of different warps and selected the best one. Zhang et al. [9] estimated reasonable seam lines by considering geometric alignment and image content, and optimized local alignment with reference to the Content Preserving Deformation (CPW) method [30]. K. Lin et al. [10] followed the mosaic line guidance, introduced contour detection and straight line detection, and used curve and straight line structures to maintain constraints in the deformation. The line matching method has obvious advantages, but the high computational complexity limits its application range. Herrmann et al. [31] made full use of object detection [32] and combined the multiple registration algorithm [11] to construct an object-centric image mosaic framework. Multiple potential planes generated by multiple registration can effectively solve the occlusion of foreground objects on the background, but it also makes the search of seam lines more complicated. In general, seam-based methods usually require high computational cost because they involve foreground and background recognition, multiple registration, seam evaluation and search, and manual interaction. They are more suitable for the situation in which there are obvious foreground objects and background in the images.

Since a global homography transformation will cause inaccurate image registration, further misalignment, and ghosting, Gao et al. proposed a dual-homography warping (DHW) model [12], which divided the scene into a background plane and a foreground plane, and used two homography matrices to align them. Since their premise was that the scene consists of two main planes, it performed well in certain specific scenes, but it could not handle more complex scenes. Lin et al. [13] proposed a smoothly varying affine (SVA) model to deal with parallax. Zaragoza et al. [14] extended this idea to a smoothly varying homography model and proposed the as-projective-as-possible (APAP) warp. The image was divided into grids, and then they used moving-DLT to calculate local adaptive homography for each grid. APAP achieves more accurate alignment in overlapping areas than DHW and has better extrapolation quality in nonoverlapping areas. Zhang et al. [26] proposed a multiviewpoint panoramic stitching method based on APAP, a local homography verification method was used to roughly align the images, and various prior constraints were used to improve the alignment through an iterative optimization scheme. Based on APAP, many works use similar methods to design more prior constraints; the combination of different constraints leads to local optimization instead of global optimization, and the computational cost is higher. Li et al. [16] proposed the elastic local alignment (ELA) and aligned images based on the Thin Plate Spline (TPS) model. The TPS function simulates the distortion of a plane based on the principle of minimum surface bending energy. It is a commonly used deformation function in biology and medical images. This function has a global nature—that is, all anchor points will have an impact on the desired point. Chen et al. [17] proposed a drone image stitching method that uses compactly supported radial basis function (CSRBF) instead of TPS to reduce local registration errors. This inspired us to think about the application of different RBFs in image stitching.

The spatially varying warping models can handle moderate parallax and provide satisfactory stitching performance, but it usually causes projective distortion outside the overlapped region. Therefore, many scholars have made further improvements. C.H. Chang et al. proposed the shape-preserving half-projective (SPHP) warps [33] from the perspective of shape correction. They made the warp gradually change from local warp to global similarity, and added similarity constraints to the entire image. Lin and Pankanti proposed an adaptive as-natural-as-possible (AANAP) warp [34], which combines a linear homography warp and a global similarity warp with minimum rotation angle, thereby creating a natural-looking mosaic. Nan Li et al. proposed a quasi-homography warp [35], which squeezes the mesh of the corresponding homography warp, but does not change its shape. These methods tried to achieve a certain balance between projection distortion and perspective distortion in nonoverlapping region, so that images can be stitched with better visual effects.

Video stitching also involves the processing of parallax, especially for videos captured by mobile cameras (e.g., smartphones or UAVs). Many researches generally perform image stitching and stabilization simultaneously to solve the ghosting and blur in the stitched video. Guo et al. [27] proposed a video stitching method acquired by two mobile handheld cameras. The intertransformation between different cameras was estimated to obtain the spatial alignment, and the intratransformation within each video was estimated to maintain the temporal smoothness. They use APAP warping method for spatial alignment to deal with parallax. Nie et al. [36] introduced a background recognition method. The backgrounds of input videos were first identified, and a seam-based strategy was used to obtain the final stitched video.

## 3. The Proposed Method

**Figure 1:**
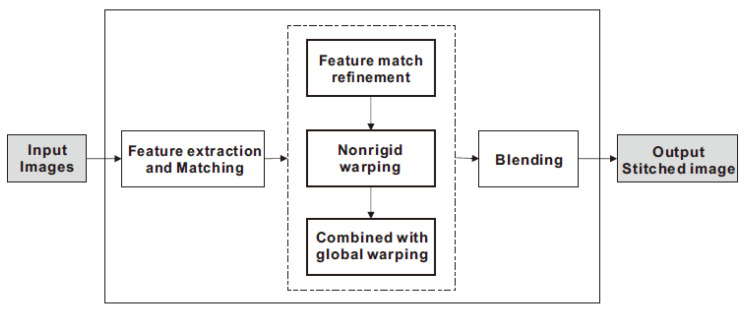
The overview flowchart of our proposed image stitching approach.

The overall workflow of our proposed image stitching is illustrated in Figure 1. First, we use the SIFT method to obtain matched feature points, and then use semiparametric fitting with motion coherence constraints to eliminate mismatches. Next, a nonrigid warp model is used to align the overlapping region. In order to maintain the shape of the nonoverlapping region, the nonrigid warping is gradually transformed into a global warping. Finally, a simple linear fusion method is used to blend the stitched images. In this section, we will give a detailed description of the feature match refinement, the nonrigid warping, and its combination with other global models.

### 3.1. Feature Match Refinement Based on Semiparametric Function Fitting

Establishing a warping model between images is the basis of stitching. When we use the method based on sparse feature points, the problem becomes to establish feature mapping relationship based on feature correspondences. Given a set of *N* putative matched features $S={\left\{\left(p_{i},q_{i}\right)\right\}}_{i=1}^{N}$ from image $I_{p}$ and $I_{q}$, $p_{i}=\left(x_{i},y_{i}\right)$ and $q_{i}=\left(u_{i},v_{i}\right)$ are two-dimensional vectors that denote the image coordinates of feature points. Our goal is to fit an appropriate function to map the coordinates from the first image to the second image, and the mapping *f* from $R^{2}$ to $R^{2}$ can be constructed as two mappings from $R^{2}$ to *R* separately—that is, $f=\left(f_{x},f_{y}\right)$, under the constraints $f_{x}\left(p_{i}\right)=u_{i}$ and $f_{y}\left(p_{i}\right)=v_{i}$ for i=1,…,N. Since parametric functions such as rigid affine or homography cannot reflect the local spatial changes of feature points, we naturally think of a smoother semiparametric function. In this article, we use a semiparametric function composed of parametric affine and nonparametric terms. Taking the *x* dimension, for example, the mapping $f_{x}$ with the input domain $p=\left(x,y\right)$ can be expressed as

(1) $$f_{x}\left(p\right)=\alpha_{1}x+\alpha_{2}y+\alpha_{3}+\phi_{x}\left(p\right)$$

where $\phi_{x}\left(p\right)$ is a smooth function with motion coherence constraint [19,37] as follows:

(2) $$\Psi_{x}=\int_{R^{2}}\frac{{\left|{\bar{\phi }}_{x}\left(\omega \right)\right|}^{2}}{\bar{g}\left(\omega \right)}d\omega$$

${\bar{\phi }}_{x}\left(.\right)$ denotes the Fourier transform of function $\phi_{x}\left(.\right)$, while $\bar{g}\left(.\right)$ is the Fourier transform of a Gaussian function $g\left(r,\sigma \right)=e^{-{\left|r\right|}^{2}/\sigma^{2}}$ with spatial distribution σ, and $\left|\cdot \right|$ denotes the Euclidean distance calculation.

In order to find the smoothest mapping, we appropriately relax the registration constraint and introduce the motion coherence constraint $\Psi_{x}$ as a regular term. This is expressed as the energy function

(3) $$E_{x}=\sum_{i=1}^{N}{\left|u_{i}-\left(\alpha_{1}x_{i}+\alpha_{2}y_{i}+\alpha_{3}+\phi_{x}\left(p_{i}\right)\right)\right|}^{2}+\lambda \Psi_{x}$$

where λ is a constant represents the weight given to the regularization term. It is difficult to directly minimize $E_{x}$ due to the existence of continuous functions $\phi_{x}\left(p\right)$ and $\Psi_{x}$. Fortunately, Andriy Myronenko et al. [37] and Wenyan Lin et al. [38] have deduced the discrete forms:

(4) $$\phi_{x}\left(p\right)=\sum_{j=1}^{N}w_{x}\left(j\right)g\left(p-p_{j},\sigma \right)=\sum_{j=1}^{N}w_{x}\left(j\right)e^{-|p-p_{j}{|}^{2}/\sigma^{2}}$$

where $g\left(p-p_{j},\sigma \right)=e^{-|p-p_{j}{|}^{2}/\sigma^{2}}$ is the Gaussian radial basis function and ${\left\{w_{x}\left(j\right)\right\}}_{j=1}^{N}$ are unknown variables;

(5) $$\Psi_{x}=w_{x}^{T}Gw_{x}$$

where $G_{N\times N}$ is a square symmetric matrix with elements $G\left(i,j\right)=g\left(p_{i}-p_{j},\sigma \right)$; it can also be called the Gaussian radial basis kernel. $w_{x}={\left[w_{x}\left(1\right),\ldots ,w_{x}\left(N\right)\right]}^{T}$ is a N×1 vector, used as the weights of the radial basis functions.

Substituting Equations (4) and (5) into Equation (3) yields

(6) $$\begin{array}{c} \arg\min_{\left\{f_{x}\left(p\right)\right\}}\sum_{i=1}^{N}{\left|u_{i}-\left(\alpha_{1}x_{i}+\alpha_{2}y_{i}+\alpha_{3}+\phi_{x}\left(p_{i}\right)\right)\right|}^{2}+\lambda \Psi_{x}\\ =\arg\min_{\left\{\alpha_{1},\alpha_{2},\alpha_{3},{\left\{w_{x}\left(j\right)\right\}}_{j=1}^{N}\right\}}\sum_{i=1}^{N}{\left|u_{i}-\left(\alpha_{1}x_{i}+\alpha_{2}y_{i}+\alpha_{3}+\sum_{j=1}^{N}w_{x}\left(j\right)g\left(p_{i}-p_{j},\sigma \right)\right)\right|}^{2}+\lambda w_{x}^{T}Gw_{x} \end{array}$$

where the energy is dependent on N+3 variables $\alpha_{1},\alpha_{2},\alpha_{3}$, and ${\left\{w_{x}\left(j\right)\right\}}_{j=1}^{N}$. Minimizing the overall energy function in Equation (6) leads to the parametrized $f_{x}$ as follows:

(7) $$f_{x}\left(p\right)=\alpha_{1}x+\alpha_{2}y+\alpha_{3}+\sum_{j=1}^{N}w_{x}\left(j\right)g\left(p-p_{j},\sigma \right)$$

The mapping from $x_{i}$, $y_{i}$ to $v_{i}$ has the similar form as

(8) $$f_{y}\left(p\right)=\beta_{1}x+\beta_{2}y+\beta_{3}+\sum_{j=1}^{N}w_{y}\left(j\right)g\left(p-p_{j},\sigma \right)$$

and the energy function is

(9) $$E_{y}=\sum_{i=1}^{N}{\left|v_{i}-\left(\beta_{1}x_{i}+\beta_{2}y_{i}+\beta_{3}+\sum_{j=1}^{N}w_{y}\left(j\right)g\left(p-p_{j},\sigma \right)\right)\right|}^{2}+\lambda w_{y}^{T}Gw_{y}$$

Since G is a positive definite matrix, the overall energy minimization problem in Equations (6) and (9) can be solved using the following linear system [39]

(10) $$\left[\begin{array}{cc} G+\lambda I & P \end{array}\right]\left[\begin{array}{cc} w_{x} & w_{y}\\ a & b \end{array}\right]=\left[\begin{array}{cc} u & v \end{array}\right]$$

where P is a N×3 matrix with the *i*th row $\left[x_{i},y_{i},1\right]$, $u={\left[u_{1},\ldots ,u_{N}\right]}^{T}$ and $v={\left[v_{1},\ldots ,v_{N}\right]}^{T}$ represent the coordinates of target points in $I_{q}$. $w_{x}={\left[w_{x}\left(1\right),\ldots ,w_{x}\left(N\right)\right]}^{T}$ and $a={\left[\alpha_{1},\alpha_{2},\alpha_{3}\right]}^{T}$, and $w_{y}={\left[w_{y}\left(1\right),\ldots ,w_{y}\left(N\right)\right]}^{T}$ and $b={\left[\beta_{1},\beta_{2},\beta_{3}\right]}^{T}$ represent two sets of pending variables.

A brief proof is as follows: Consider the energy function $E_{x}$ in Equation (6). The matrix form of its derivative with respect to $w_{x}$ should be zero.

(11) $$\frac{\partial E_{x}}{\partial w_{x}}=2G\left(\left(Gw_{x}+\mathrm{Pa}\right)-u\right)+2\lambda Gw_{x}=0$$

Multiplying Equation (11) by $1/2G^{-1}$, we obtain

(12) $$\left(G+\lambda I\right)w_{x}+\mathrm{Pa}=u$$

Through the above analysis, we successfully treat image warping as a general matching problem with smoothness constraint. Each feature point has its own associated mapping parameters, rather than all points sharing the same set of parameters. $f_{x}$ and $f_{y}$ can be regarded as a pair of smooth surface fitting functions. We transform the smooth function into the sum of a finite number of radial basis functions, so that the problem of minimizing a convex cost function is transformed into solving a linear system. After each solution, we use the median absolute deviation (MAD) method [40] to remove outliers—that is, the points whose deviation from the fitted function is lager than 1.48 times MAD will be regarded as outliers. The mapping parameters are recalculated using inliers, and this process repeats three times. This is a more flexible feature match refinement method that can reserve more points from a set of initial correspondences based on feature descriptors.

### 3.2. Nonrigid Warping Based on Gaussian Radial Basis Functions

As mentioned before, the smooth function $\phi \left(.\right)$ has a discrete form in Equation (4). It is a linear sum of Gaussian radial basis functions (GRBFs). They are constructed based on the Euclidean distances between the target point to the control points. Taking $I_{q\;}$ as the reference image, we transform the coordinates (x,y) of an arbitrary point in image $I_{p}$ into the coordinate system of the reference image to become $\left(x^{\prime },y^{\prime }\right)$. The feature points ${\left\{p_{i}=\left(x_{i},y_{i}\right)\right\}}_{i=1}^{M},M\leq N$ in image $I_{p}$ are used as the control points, and their correspondences in image $I_{q}$ are ${\left\{q_{i}=\left(u_{i},v_{i}\right)\right\}}_{i=1}^{M}$. Our nonrigid warp model has the same form as the feature mapping function, which is a polynomial plus the linear sum of GRBFs in each dimension. The formula is as follows:

(13) $$\left\{\begin{array}{c} x^{\prime }=f_{x}\left(x,y\right)=\alpha_{1}x+\alpha_{2}y+\alpha_{3}+\sum_{i=1}^{M}w_{x}\left(i\right)e^{-|\left(x,y\right)-p_{i}{|}^{2}/\sigma^{2}}\\ y^{\prime }=f_{y}\left(x,y\right)=\beta_{1}x+\beta_{2}y+\beta_{3}+\sum_{i=1}^{M}v_{x}\left(i\right)e^{-|\left(x,y\right)-p_{i}{|}^{2}/\sigma^{2}} \end{array}\right.$$

where $\left\{w_{x}\left(1\right),\ldots ,w_{x}\left(M\right),\alpha_{1},\alpha_{2},\alpha_{3}\right\}$, and $\left\{w_{y}\left(1\right),\ldots ,w_{y}\left(M\right),\beta_{1},\beta_{2},\beta_{3}\right\}$ are variables calculated based a set of *M* features correspondences after match refinement. The linear system is also used in Equation (10).

**Figure 2:**
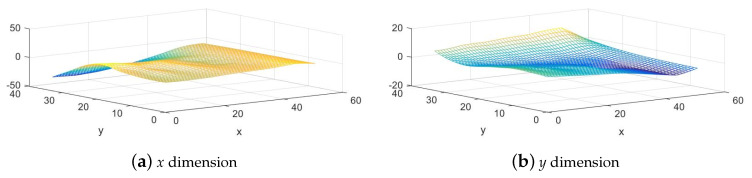
Nonrigid warp using mesh deformation.

In order to better apply nonrigid warp to image stitching, it is necessary to overcome the large amount of calculation, because, for our nonrigid warp model, each pixel on the target image has its own deformation parameters, and its Euclidean distances to all control points need to be calculated. Therefore, the pixel-by-pixel calculation will be time-consuming. We use mesh deformation to speed up the calculation: before resampling, we divide the image into a grid mesh of $C_{1}\times C_{2}$ cells, calculate the deformation on the grid nodes first, and then obtain other points’ coordinates through linear interpolation. A visualization example in Figure 2 shows how the image is warped based on our nonrigid warp model and mesh deformation. The parallax between pixels is regarded as the “height” above the image plane in the imaginary third dimension. Then, the smooth surface fitted by the nonrigid model is reprojected to the reference image plane. Therefore, our method is suitable for image stitching with smooth varying parallax.

### 3.3. Smooth Transition to Global Warping

In addition to warping, image stitching also involves extrapolating the warp model calculated based on the overlapping region to the nonoverlapping region. Because of the strong intervention of the matching points, the nonrigid warp leads to better alignment in the overlapping region. However, if it is forced to extrapolate to the nonoverlapping region, this part of the image will be excessively distorted. Therefore, we choose a common global warp (such as similarity and homography warps) to maintain the shape of the image in nonoverlapping regions.

According to the given feature correspondences, the least squares method is usually used to minimize the projection error of all feature points to solve a global warp model for image stitching, and it is easy to implement using the Levenberg–Marquardt (LM) algorithm [41].

Similarity warp describes the rotation, translation, and scaling of the image, and its matrix form is as follows:

(14) $$S=\left[\begin{array}{ccc} s\cos(\theta ) & -s\sin(\theta ) & t_{x}\\ s\sin(\theta ) & s\cos(\theta ) & t_{y}\\ 0 & 0 & 1 \end{array}\right]$$

The corresponding coordinate transformation is:

(15) $$\left\{\begin{array}{c} x^{\prime }=S_{x}\left(x,y\right)=s\cos\left(\theta \right)x-s\sin\left(\theta \right)y+t_{x}\\ y^{\prime }=S_{y}\left(x,y\right)=s\sin\left(\theta \right)x+s\cos\left(\theta \right)y+t_{y} \end{array}\right.$$

The homography warp, also known as the perspective transformation, has the matrix form as follows:

(16) $$H=\left[\begin{array}{ccc} h_{00} & h_{01} & h_{02}\\ h_{10} & h_{11} & h_{12}\\ h_{20} & h_{21} & 1 \end{array}\right]$$

The corresponding coordinate transformation is:

(17) $$\left\{\begin{array}{c} x^{\prime }=H_{x}\left(x,y\right)=\frac{h_{00}x+h_{01}y+h_{02}}{h_{20}x+h_{21}y+1}\\ y^{\prime }=H_{y}\left(x,y\right)=\frac{h_{10}x+h_{11}y+h_{12}}{h_{20}x+h_{21}y+1} \end{array}\right.$$

A simple method is to directly set the nonrigid warp of the nonoverlapping region to zero, but it will cause a sudden change in the overlapping edge. In this paper, the nonrigid warp is gradually reduced to achieve a smooth transition. As the point p(x,y) gradually moves away from the overlapping area, the scale parameter ε gradually changes from 1 to 0 (taking nonrigid warp + similarity warp, for example):

(18) $$\left\{\begin{array}{c} x^{\prime }=\varepsilon f_{x}\left(x,y\right)+\left(1-\varepsilon \right)S_{x}\\ y^{\prime }=\varepsilon f_{y}\left(x,y\right)+\left(1-\varepsilon \right)S_{y} \end{array}\right.$$

ε is calculated as:

(19) $$\varepsilon =\left\{\begin{array}{cc} 1 & ,W_{s}\leq 0\\ 1-W_{s}/W_{b} & ,0<W_{s}\leq W_{b}\\ 0 & ,W_{b}<W_{s} \end{array}\right.$$

where $W_{s}=\max\left(x-x_{b},x_{a}-x,y-y_{b},y_{a}-y\right)$, $W_{b}=\eta *\min\left(x_{b}-x_{a},y_{b}-y_{a}\right)$. $\left[x_{a},x_{b}\right],\left[y_{a},y_{b}\right]$ are the coordinate ranges in *x* and *y* directions of the overlapped region calculated by the global warp, and η is a constant used to control the width of the transition area.

**Figure 3:**
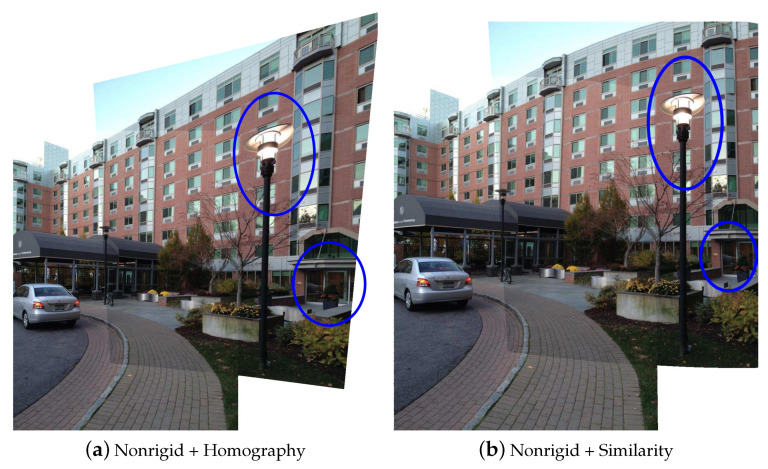
Combination with different global models. The visual effect of homography warp is not as good as similarity warp, because the streetlight and door in nonoverlapping region are severely deformed.

A comparison of using different global warps is illustrated in Figure 3, using image pair “building” from [34]. The main difference is in nonoverlapping region. Homography warp preserves all straight lines, but the region of an object is enlarged or stretched compared to its appearance in the original image. Similarity warp preserves the original shape of the object, since they purely involve translation, rotation, and uniformly scaling, but the perspectives of an object in two images may be inconsistent with each other [35]. In Figure 3a, the streetlight and door are stretched. In Figure 3b, the streetlight maintains its original shape, but the shape of the top of the building changes from straight to slightly curved. When stitching wide-field-of-view images, homography may cause large projective distortion in the nonoverlapping region, which is inconsistent with human cognitive habits. So we prefer to use a similarity warp to maintain the shape of nonoverlapping region. This is consistent with the conclusion of the SPHP [33] method, which also used similarity transformation in the nonoverlapping region.

## 4. Experiments and Discussion

We compare our nonrigid warp against the global homography warp and other two spatially varying warps for image stitching—that is, as-projective-as-possible (APAP) warp [14] and elastic local alignment (ELA) [16]. The SIFT [23] method is used to provide initial feature correspondences. In order to cogently evaluate these methods, we simply blend the aligned images by pixel intensity averaging so that any misalignment remain obvious. The image data sets includes several urban scene images from other related works.

The experiments are performed on a laptop with Intel i7 CPU@2.70GHz and Matlab Code.

### 4.1. Parameter Settings

The semiparametric function and the nonrigid warp model involve two free parameters λ and σ. λ represents the trade-off between the feature registration and the smoothness constrain, and σ reflects the strength of the interaction between the feature points. A larger σ will lead to a flatter warping, and the same is to λ. In order to take into account both the image size and the distribution of feature points, we set σ=100×(ho+wo)/ptnum, where ho and wo represent the height and width of the overlapped region and ptnum represents the number of feature points. λ is set to be π/3 correspondingly.

**Figure 4:**
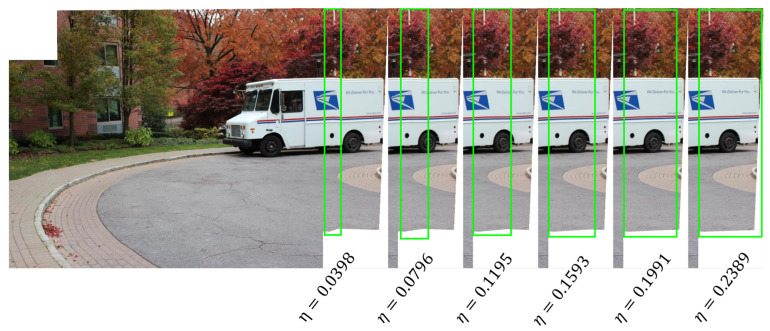
Different transition effects with different η. The green boxes represent the approximate transition region. A small η will cause an unnatural transition, so we try to make the transition area cover the nonoverlapping region on the right side.

The constant η used in smooth transition is an empirical value. It acts as a scale parameter of the width of overlapping region wo. The larger the η, the larger the width of the transition area. Let the width of a single image be wi. After experimenting with multiple sets of images, we suggest setting η by making $W_{b}=1.5\times \left(wi-wo\right)$. Figure 4 shows a set of stitched results of image pair “roundabout” [34], where $W_{b}$ are 0.25, 0.5, 0.75, 1.0, 1.25, and 1.5 times of (wi−wo), respectively, and the corresponding η are 0.0398, 0.0796, 0.1195, 0.1593, 0.1991, and 0.2389.

**Figure 5:**
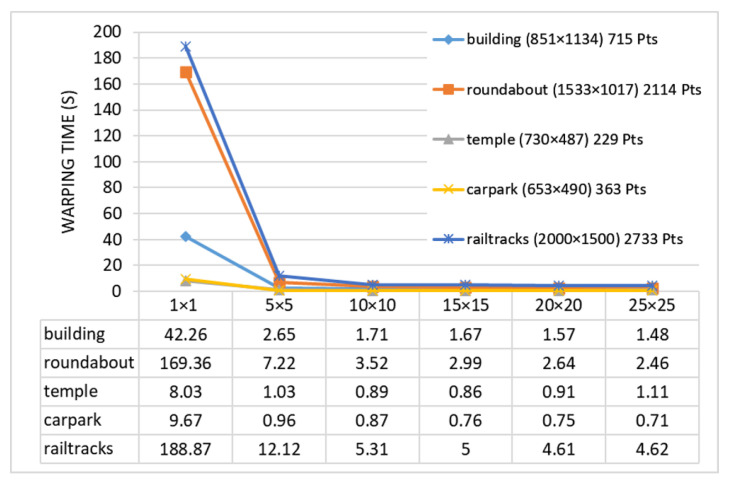
Warping time statistics for different grid sizes. The selected images have different image size and different number of feature points. For each grid size, we count the warping time 10 times and take the average time.

In the mesh deformation stage, the larger the grid cell, the shorter the calculation time, but the precision of model fitting will also decrease. Therefore, it is necessary to find a suitable grid size to balance efficiency and stitching quality. We selected five pairs of images to count the deformation time corresponding to different grid sizes. The results are shown in Figure 5, where the result of 1×1 pixel is the time it takes for pixel-by-pixel deformation. It can be seen from the chart that when the grid size is 5×5 pixels, the warping time has been greatly shortened, but the acceleration effect of larger grid sizes such as 20×20 pixels and 25×25 pixels is no longer obvious. In our experiments, the size of the grid cell is set to 10×10 pixels.

### 4.2. Qualitative Comparisons

**Figure 6:**
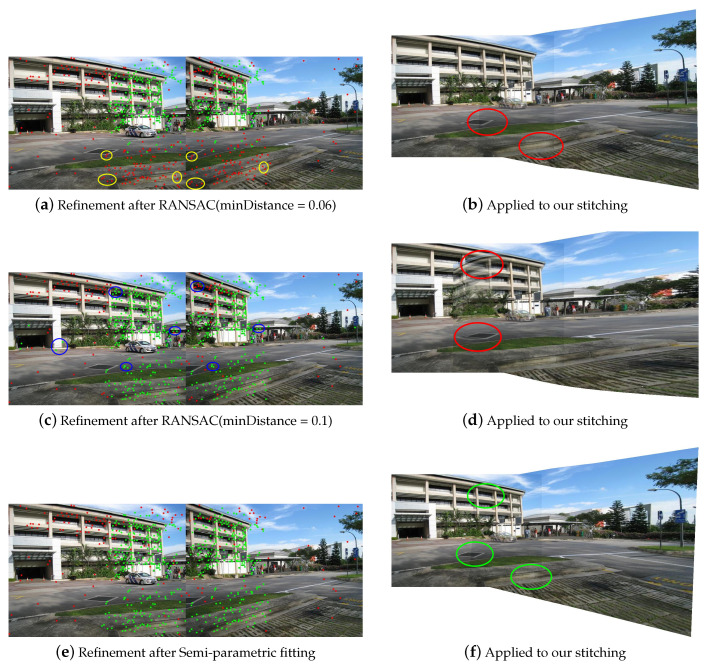
Feature match refinement. (**a**,**c**) are the outlier removal results of global RANSAC. The green dots and the red dots indicate the retained and removed features, respectively. Some of the removed inliers are marked in yellow, while the remaining outliers are marked in blue. (**b**,**d**) are the stitching results of our nonrigid warp based on the global RANSAC results. (**e**) is the feature refinement result of our semiparametric fitting. (**f**) is the stitching result of our method.

First of all, our feature match refinement is compared with the traditional RANSAC method. Homography is selected as the global model to be fitted for RANSAC. The maximum number of iterations in the experiments is set to 500; this determines the computational efficiency of RANSAC algorithm and has been proved to be a reliable empirical value in the APAP [14] and ELA [16] methods. Another threshold minDistance is used to determine the feature points that are fit well by global model, which directly determines the number and distribution of matched features. With a smaller minDistance, some inliers may be eliminated because they do not conform to the fitted global model, as shown in Figure 6a. With a larger minDistance, some outliers will be retained, which are not conducive to our nonrigid warping, as shown in Figure 6c,d. Figure 6e,f show that our semiparametric fitting method can retain more matched features, thereby producing better stitching results.

**Figure 7:**
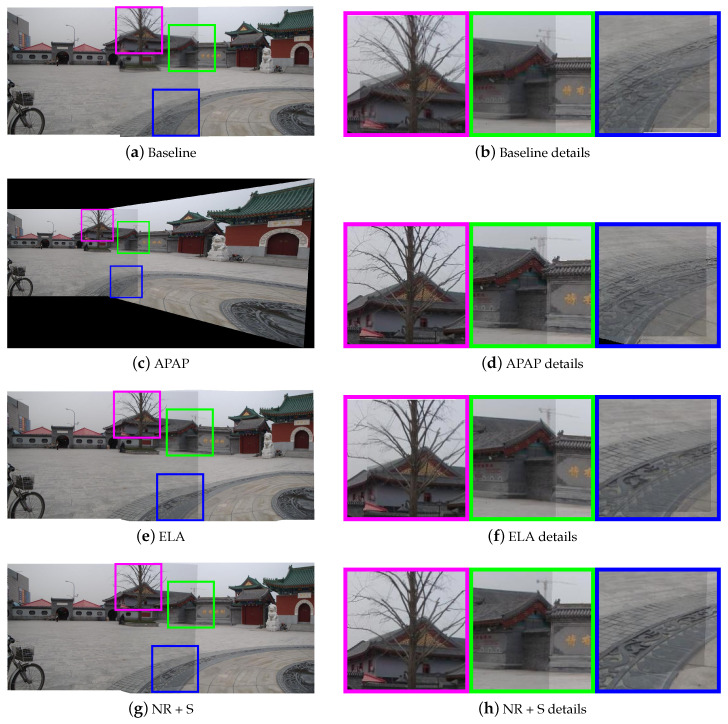
Comparison of stitching quality on “temple”. (**a**,**b**) are results of the baseline with obvious local misalignments. (**c**,**d**) are results of as-projective-as-possible (APAP). (**e**,**f**) are resluts of elastic local alignment (ELA). (**g**,**h**) are resluts of nonrigid + similarity warping.

**Figure 8:**
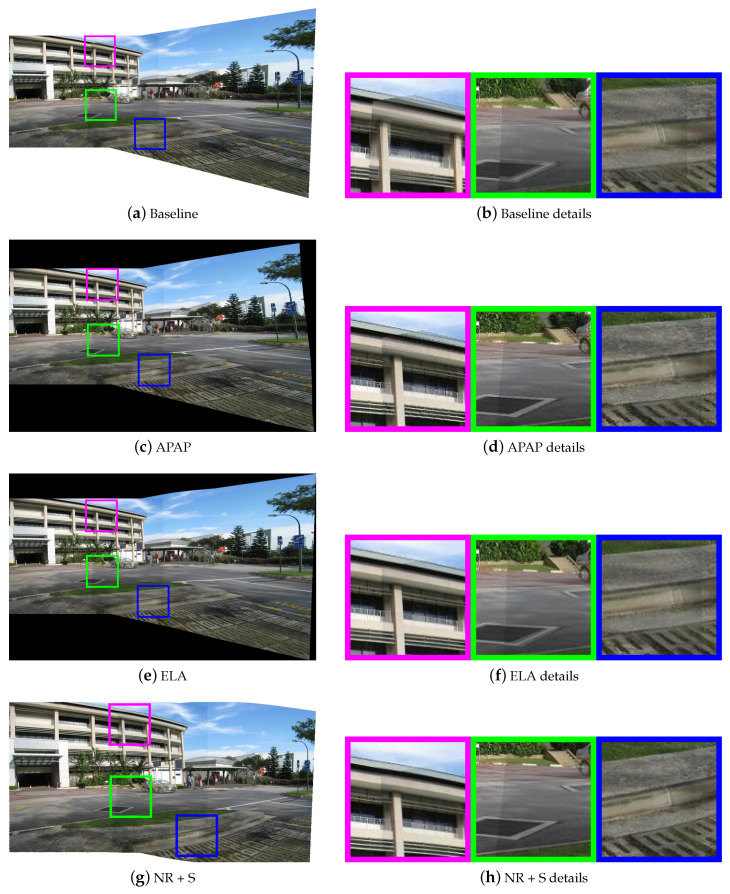
Comparison of stitching quality on “carpark”. (**a**,**b**) are results of the baseline with obvious local misalignments. (**c**,**d**) are results of APAP. (**e**,**f**) are results of ELA. (**g**,**h**) are results of nonrigid + similarity warping.

**Figure 9:**
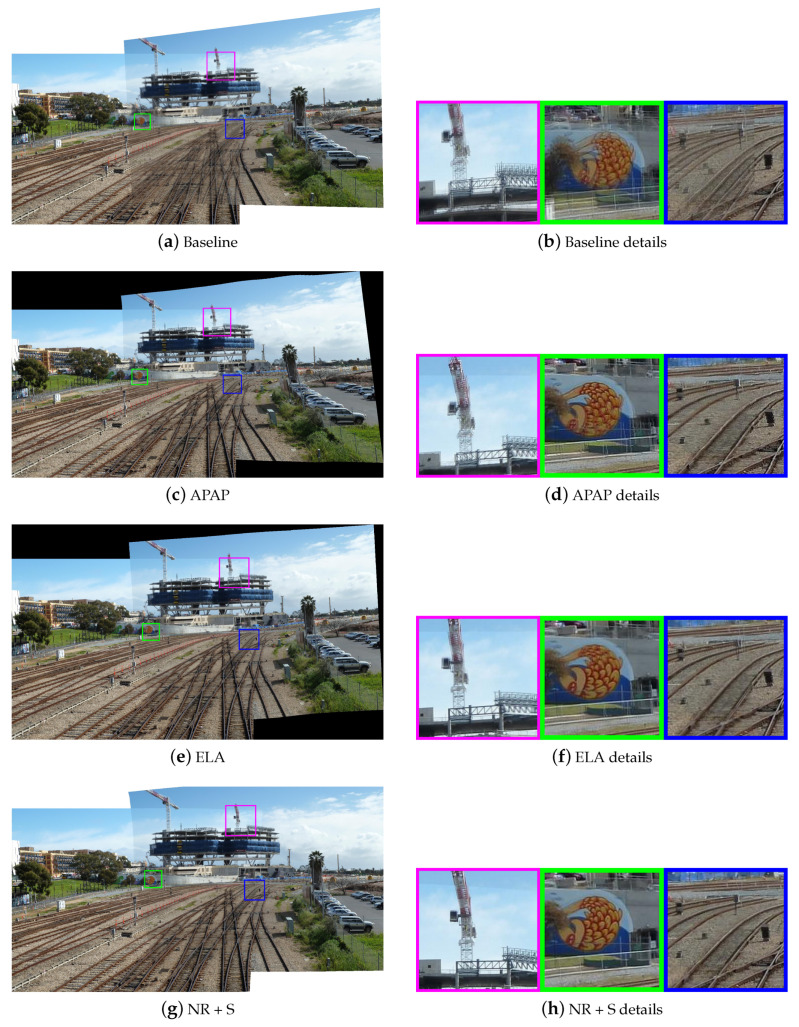
Comparison of stitching quality on “railtrack”. (**a**,**b**) are results of the baseline with obvious local misalignments. (**c**,**d**) are results of APAP. (**e**,**f**) are results of ELA. (**g**,**h**) are results of nonrigid + similarity warping.

Then, we selected three pairs of urban scene images to visually demonstrate the stitching quality of various methods. They are “temple” from AANAP [34], “carpark” from DHW [12], and “railtrack” from APAP [14]. Figure 7, Figure 8 and Figure 9 show the results of different methods, three representative regions of each resulting image are highlighted. The first row shows the results of the global homography, which serves as the baseline for comparison, with obvious misalignment in all highlighted areas. The second row shows the results of APAP, the third row shows the results of ELA, and the fourth row shows the result of our nonrigid warp combined with a global similarity warp.

Figure 7 is a case of image pairs with low overlap. In the result of APAP, the nonoverlapping region of the image is severely stretched, and ghost still exists in the regions marked in green and blue. In the result of ELA, the ground suffered some distortion around the overlapping borders. In all marked regions, our nonrigid warp achieves a more accurate local alignment. Compared to the ELA method, our transition to nonoverlapping region is smoother. In Figure 8, the scene can be clearly divided into the background and the ground. In the green marked regions, APAP aligns the manhole cover on the ground, but fails to align the steps in the background, and ELA’s results are exactly the opposite. Our nonrigid warp is more flexible to align the background and ground simultaneously. Figure 9 shows a challenging data set used in APAP which is rich of complex curved features. Our nonrigid warp also has a good performance and can successfully align railways and rod-shaped objects. The marked regions show better alignments than APAP and ELA methods.

In general, our nonrigid warp performs better in the selected data sets. The nonrigid warping leads to more accurate local alignment. The combination with similarity warps leads to better visual effects in non-overlapping region.

### 4.3. Quantitative Comparisons

To quantify the alignment accuracy of our nonrigid warp $f=\left\{f_{x},f_{y}\right\}:R^{2}\mapsto R^{2}$, we compute the root mean squared error (RMSE) of *f* on a set of corresponding feature points ${\left\{{\left(x,y\right)}_{i},{\left(x^{\prime },y^{\prime }\right)}_{i}\right\}}_{i=1}^{M}$, where $\mathrm{RMSE}\left(f\right)=\sqrt{\frac{1}{M}\sum_{i=1}^{M}\left({\left|f_{x}\left(x_{i},y_{i}\right)-x_{i}^{\prime }\right|}^{2}+{\left|f_{y}\left(x_{i},y_{i}\right)-y_{i}^{\prime }\right|}^{2}\right)}$.

In addition to the matched feature points used for the calculation of the warp model, we also manually selected 20–30 groups of uniformly distributed checkpoints, and also counted their root mean square errors. For each pair of images, we repeat the statistics 20 times, and then use the average of the results. Table 1 shows the REMSs of feature points and checkpoints, corresponding to homography warp, APAP, ELA and our nonrigid warp respectively. Compared with other methods, nonrigid warp can obtain smaller REMSs (shown in bold), which means our method has higher alignment accuracy.

### 4.4. Limitations

**Figure 10:**
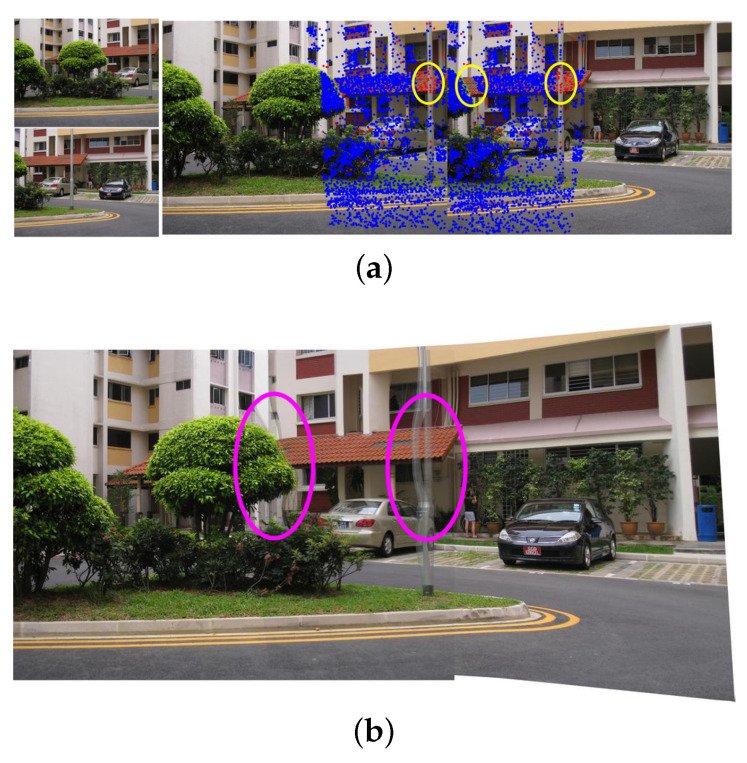
A failure case using our method. (**a**) Occlusions will cause the lack of feature points. (**b**) The artifacts are circled in the stitched image.

The proposed nonrigid warping fits the parallax in a similar way to smooth surface fitting. Occlusions in the images will cause discontinuous changes in depth differences and make the occluded parts lack matched features. Therefore, if there are severe occlusions in the images, our method will be powerless. Figure 10 shows a failure case in which ghosting effects appear around the foreground objects. Similar to other spatially varying alignment methods, straight lines will be curved in order to achieve precise local alignment. For applications that need to preserve straight lines, adding line features may be helpful. Another limitation of our method is effectiveness. In the implementation of the experiment, the ELA method is faster than APAP, and our method is somewhere in between. The main time cost is in the step of feature match refinement. In the case of sufficient and well-matched features, an effective speed-up method is to skip the semiparametric fitting and directly calculate the nonrigid warp model.

## 5. Conclusions

In this paper, we propose an effective image stitching method based on nonrigid warping. First, the semiparametric functions fitting is used to refine the features matched by descriptions. This new feature mapping relationship provides more feature points and helps to eliminate the influence of parallax. Second, a nonrigid warp model based on the Gaussian radial basis functions is derived from the semiparametric functions, and a uniform grid is set on the image plane to speed up the calculation of the warping. As a kind of surface fitting model, the proposed nonrigid warp can adapt to the spatial change of the projection relationship. This results in a more precise alignment in the overlapping region of the image. Finally, the nonrigid warp is effectively combined with a global transformation to improve local alignment while reducing distortion in nonoverlapping regions. The stitching quality of our method is evaluated through several comparative experiments. Our method has good performance in both visual effects and accuracy. In terms of quality, there is less blur and ghost in our stitching results. In terms of quantity, the projection error of feature points using our nonrigid warping is smaller than that of feature points using ELA and APAP methods. In future work, we will try to improve efficiency of our method, and the proposed nonrigid warp model will be applied to aerial image stitching to eliminate the parallax caused by terrain fluctuations.

## Figures and Tables

**Table 1:** Comparison of average root mean squared error RMSE for the proposed method and other methods.

Image Pair		Number	Baseline	APAP	ELA	Nonrigid
temple [34]	matches	-	3.98	2.51	0.88	**0.35**
	checkpoints	25	3.34	2.18	1.33	**1.24**
carpark [12]	matches	-	4.71	2.10	1.70	**1.084**
	checkpoints	24	6.06	1.85	4.98	**0.89**
railtracks [14]	matches	-	14.54	4.70	4.10	**1.28**
	checkpoints	21	21.54	1.80	2.29	**1.81**
building [34]	matches	-	3.66	4.33	2.81	**1.79**
	checkpoints	23	3.2	1.59	2.15	**1.76**
